# Supplementary material for: Phage on tap–a quick and efficient protocol for the preparation of bacteriophage laboratory stocks
Source: PeerJ. 2016 Jul 26;4:e2261. doi: 10.7717/peerj.2261 (PMC4975003; doi:10.7717/peerj.2261)
Supplement: Supplemental Information 1 — Materials and Methods, Figs. S1 and S2. [file peerj-04-2261-s001.docx]

Supporting Online Material for

**Phage on Tap – A quick and efficient method for the preparation of bacteriophage laboratory stocks**

Natasha Bonilla^1^*^*^*, Maria Isabel Rojas^1^, Giuliano Netto Flores Cruz^1^, Shr-Hau Hung^1^, Forest Rohwer^1^, and Jeremy J. Barr^1^

^1^Department of Biology, Department of Biology, San Diego State University, 5500 Campanile Dr, San Diego, CA 92182-4614, USA

^*^To whom correspondence should be addressed. Email: jeremybarr85@gmail.com

**This PDF file includes**

Materials and Methods

Figures S1-S2

**Materials and Methods**

**Traditional Method for Phage Purification and Concentration By Plate Lysate (Fig. S1.)**

**Phage Isolation and Amplification from a Single Plaque**

A single plaque of the phage (“plug”) was transferred directly with a sterile Pasteur pipet from the plate that it was initially detected to generate homogeneous stocks. The “plug” was resuspended into a microcentrifuge tube with 700 μl of filter-sterilized Suspension Medium (SM) buffer (SM per liter: 5.8 g NaCl, 2.0 g MgSO_4_.7H_2_O, 50 ml 1M Tris-HCl pH 7.4). Suspension was then centrifuged at 1,500 x g for 20 min to remove any remaining debris. The supernatant was stored at 4 °C and titered via top agars as described below.

**Phage Plaque Assay (or “Top Agars”)**

To determine the phage titer, phage plaque assays (or “top agars”) were performed using Luria-Bertani (LB) broth (10 g Tryptone, 5 g Yeast Extract, 10 g NaCl, in 1 liter dH_2_O), LB top agar (25 g LB broth, 7.5 g Agar, in 1 liter dH_2_0) and LB agar plates (25 g LB broth, 15 g Agar, in 1 liter dH_2_0) (BD Biosciences). Stocks of T4 phages were serially diluted in 1 ml LB broth. Briefly, dilutions were mixed with 3 ml of LB top agar and 1 ml *Escherichia coli* B strain and then poured onto LB agar plates. Plates were incubated at 37 °C and viral plaques were counted after 24 hrs of incubation.

**Phage Harvesting: Plate Lysate**

Approximately, 5 ml of 0.02 μm sterile syringe filtered (WhatmanTM, AnotopTM 25) SM Buffer was added to those plates showing complete viral lysis and slowly agitated rotationally for 10 min. The SM buffer was transferred to a sterile conical centrifuge tube (BD Biosciences) and then centrifuged at 4,000 x g for 5 min at 25 ºC.

**Phage Clean-Up: 0.22 μm Filtering and Chloroform Treatment**

The supernatant was 0.22 μm sterile syringe filtered (Millipore, Billerica, MA, USA) to yield a bacterial cell-free phage stock. 0.1 volumes of chloroform (Fisher Scientific) was added to the supernatant containing the viruses (aqueous layer), gently vortexed and then centrifuged at 4,000 x g for 5 min at 25 ºC. The supernatant was transferred into properly labeled sterile falcon centrifuge tubes. PFU per ml was quantified via top agar assay as described above to ensure an initial high phage titer (>1×10^9^ PFU·ml^-1^).

**Phage Purification Using Cesium Chloride Density Gradients**

Cesium chloride (CsCl) ultracentrifugation was used to obtain a concentrated, pure phage stock through high-speed density gradient separation (**Fig. S2**.) Approximately 8 ml of each phage concentrate was centrifuged atop of a CsCl gradient (Fisher Scientific), at 50,000 x g at 4 ºC for 2 hrs in SW41 Ti rotor. The phage concentrate formed a grey-white band in the 1.5 g·ml^-1^ density layer of the centrifuge tube (Beckman Coulter, Palo Alto, CA, USA). The phage band (~1.5 ml) was carefully collected by inserting a syringe with an 18 ga needle (BD, Franklin Lakes, NJ, USA) through the wall of the tube, 2-3 mm below the 1.7 g·ml^-1^ density band (red arrow in **Fig.S2.**), and transferred into a sterile eppendorf tube. At this step phage plaques were again quantified via top agar assay to ensure phage were not lost.

**Dialysis**

CsCl was removed from the phage suspensions by dialysis against 2 liters of SM buffer at 4 ºC with stirring for 24 hrs. CsCl purified phage was transferred into 10 ml Spectra-Por^®^ Float-A-Lyzer^®^ G2 Dialysis membrane (10 mL, MWCO 3.5-5 kDa, Sigma-Alrich, USA) using a sterile syringe and 21 G needle (BD Bioscience). The SM buffer was replaced after 12 hrs and the salinity of the concentrating solution was measured using a refractometer to ensure sufficient cesium salts were removed from phage sample (**Fig. S3**). After the completion of dialysis, the phage preparation was carefully removed from the cassette using a sterile syringe and 21 G needle (BD Bioscience) and PFU·ml^-1^ was quantified via top agar assay, as described above. Details for the insertion and withdrawing of the sample can be found on the manufacturer’s instructions.

**Phage Storage**

Phage stocks were titered in duplicate over a three moth period (**Fig. S4**). Phage were aliquoted into cryovials and stored at five different conditions (duplicates per condition): 4 °C + SM buffer, -20 °C + 50% v/v glycerol, -80 °C+ 50% v/v glycerol, liquid nitrogen with 50% v/v glycerol (Fisher Scientific) and liquid nitrogen with 5% v/v Dimethyl sulfoxide (DMSO) (Sigma Aldrich).

**Transmission Electron Microscopy (TEM) analysis**

The grids containing the phage concentrates were rinsed 3 times with 20 μl of water to remove salts from the SM storage buffer and negatively stained by uranyl acetate (0.5 %) for 15 seconds, dried, and analyzed using the FEI Tecnai T12 TEM (FEI, Hillsboro, OR) at the San Diego State University Electron Microscopy Facility.

**Supporting Online Figures**


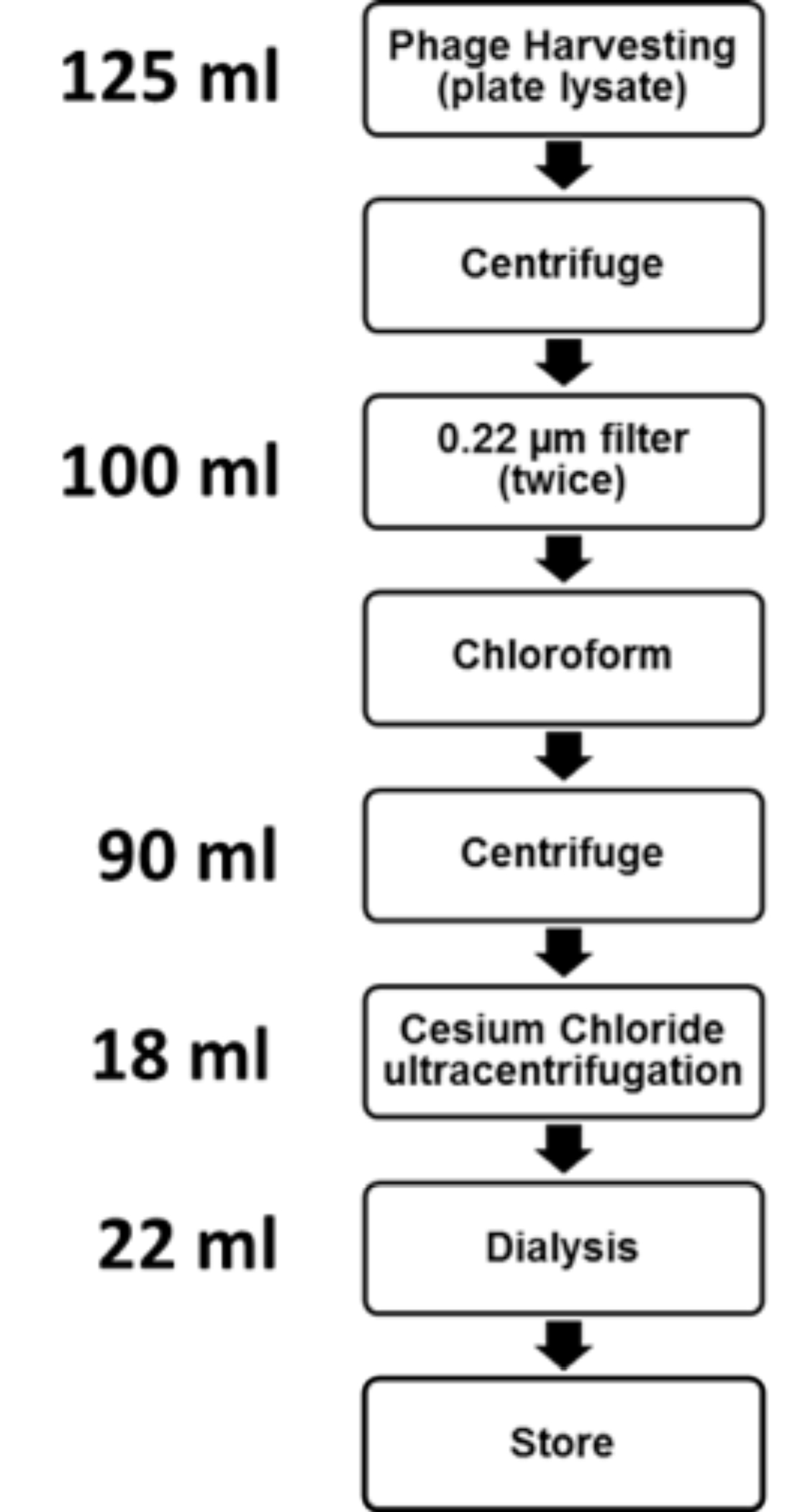


**Figure S1.** Flow chart of the traditional method for the production of phage lysates by top agar propagation, centrifugation, filtering, chloroform treatment, CsCl ultracentrifugation, extraction of CsCl band, and desalting through dialysis. Titration of phage suspensions was determined after each step by performing top agar methods. Volume (ml) was recorded after each step. This process takes up to five days.


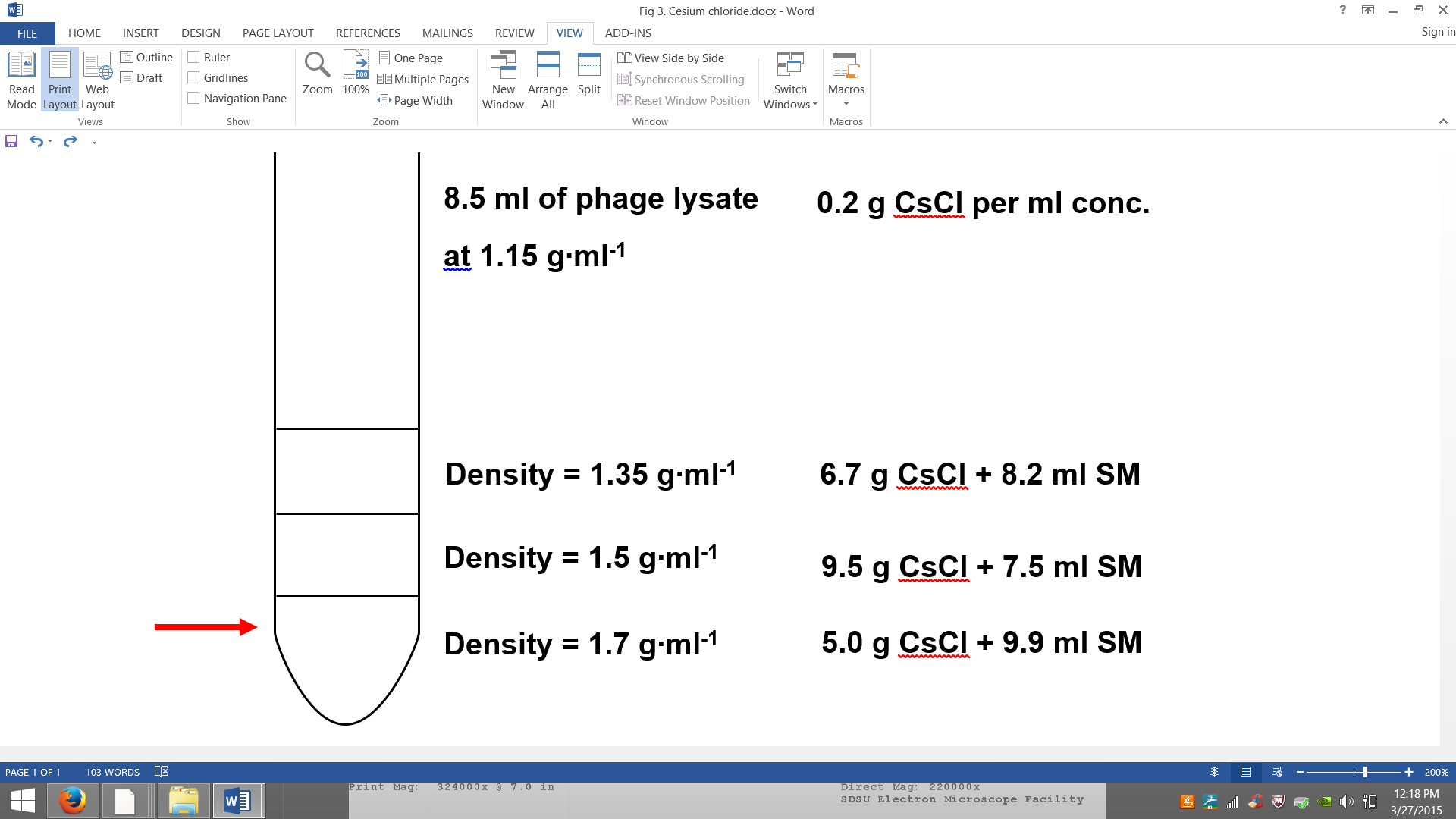


Figure S2. Phage purification by centrifugation in cesium chloride (CsCl) gradients. Phages were concentrated in a four-layer gradient of different concentrations of CsCl (1.15 g∙ml^-1^, 1.35 g∙ml^-1^, 1.5 g∙ml^-1^, 1.7 g∙ml^-1^). A sterile 18-ga needle was inserted just before the 1.5 g∙ml^-1^ density to remove the concentrated phages (see red arrow).


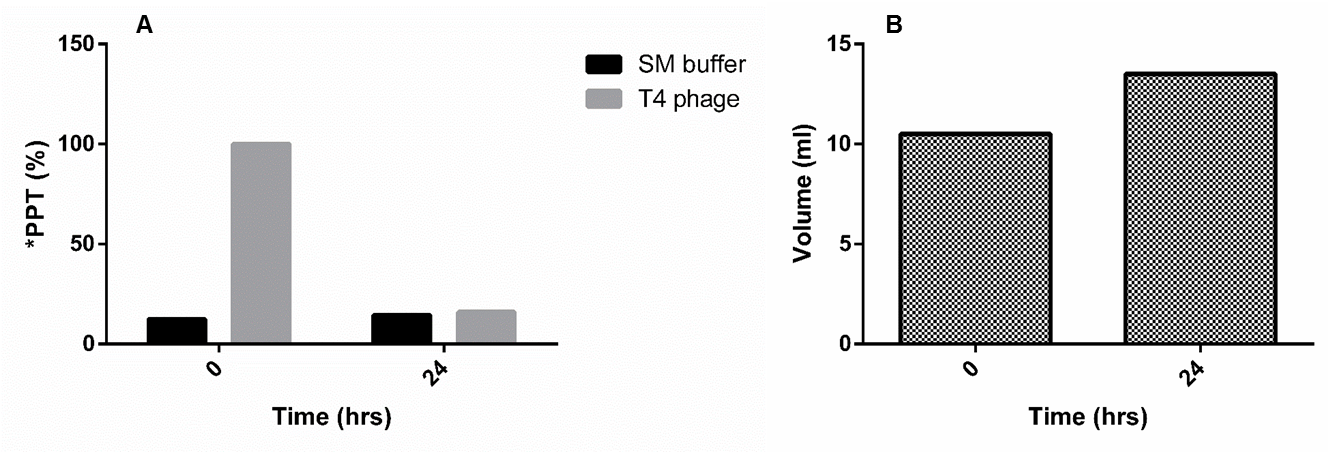


Figure S3. Refractometer data showing the removal of cesium chloride salts from traditional phage preparations. (A) Salinity (%) was reduced after 24 hrs dialysis against 2 liters of SM buffer for 24 hrs in the cold. (B) The initial volume (ml) of T4 phage lysate slightly increased.


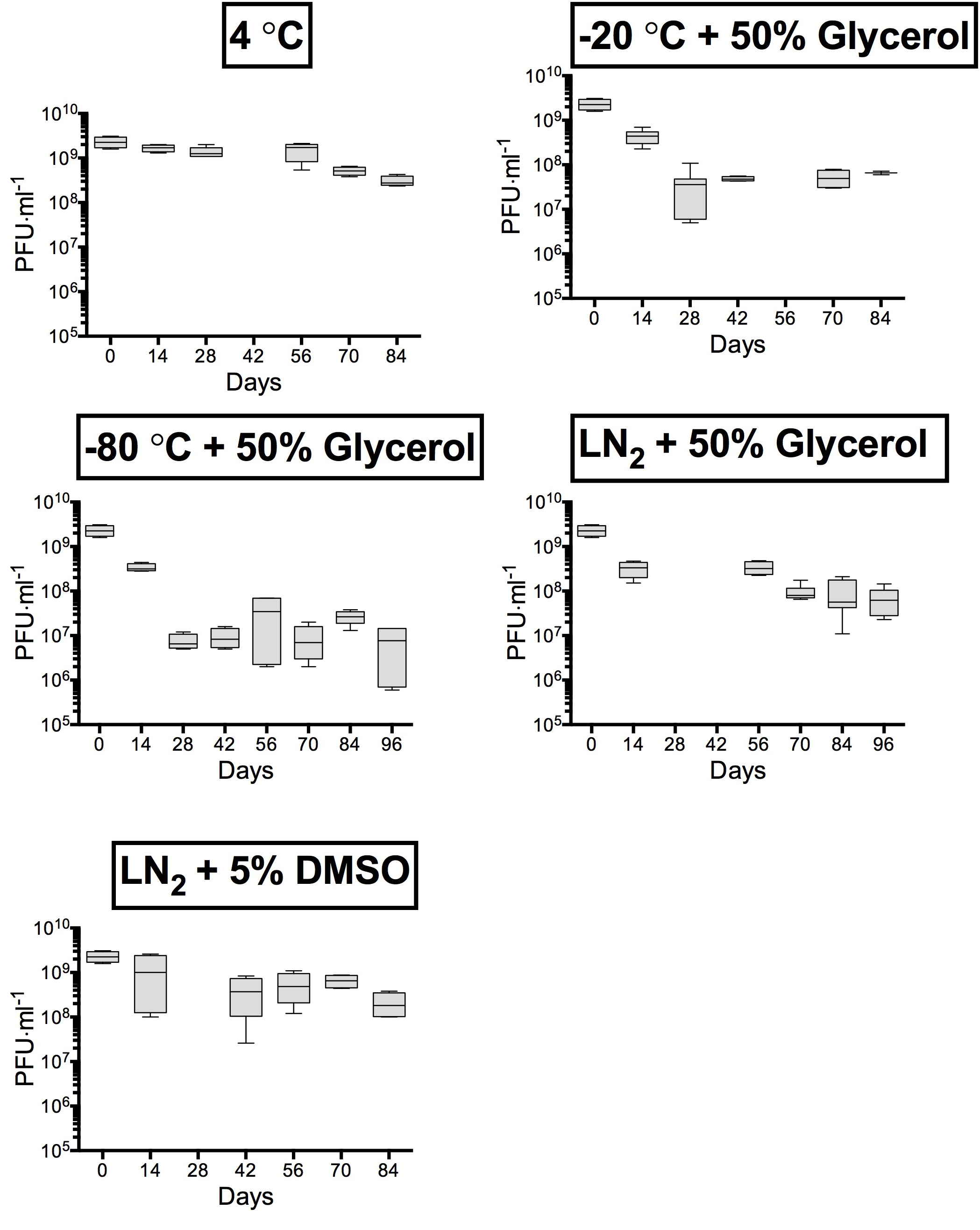


**Fig. S4**. Phage stability counts as measured by top agar of phage concentrates stored at 4 °C in SM buffer, liquid nitrogen (LN_2_) in 5% (v/v) DMSO, LN_2_ in 50% (v/v) glycerol, -20 °C in 50% glycerol and -80 °C in 50% glycerol.
